# ELIXIR-IT HPC@CINECA: high performance computing resources for the bioinformatics community

**DOI:** 10.1186/s12859-020-03565-8

**Published:** 2020-08-21

**Authors:** Tiziana Castrignanò, Silvia Gioiosa, Tiziano Flati, Mirko Cestari, Ernesto Picardi, Matteo Chiara, Maddalena Fratelli, Stefano Amente, Marco Cirilli, Marco Antonio Tangaro, Giovanni Chillemi, Graziano Pesole, Federico Zambelli

**Affiliations:** 1 Department of Ecological and Biological Sciences (DEB), University of Tuscia, Viterbo, Italy; 2 CINECA, SuperComputing Applications and Innovation Department, Rome, Italy; 3 Institute of Biomembranes, Bioenergetics and Molecular Biotechnologies, National Research Council (IBIOM-CNR), Bari, Italy; 4 Department of Biosciences, Biotechnology and Biopharmaceutics, University of Bari “A. Moro”, Bari, Italy; 5 Department of Biosciences, University of Milan, Milan, Italy; 6 IRCCS-Istituto di Ricerche Farmacologiche “Mario Negri”, Milano, Milan, Italy; 7 Department of Molecular Medicine and Medical Biotechnologies, University of Naples ‘Federico II’, Naples, Italy; 8 Department of Agricultural and Environmental Sciences - Production, Landscape, Agroenergy (DISAA), University of Milan, Milan, Italy; 9 Department for Innovation in Biological, Agro-food and Forest systems (DIBAF), University of Tuscia, Viterbo, Italy

**Keywords:** HPC, Compute service, Bioinformatics, Software environment, NGS data analysis

## Abstract

**Background:**

The advent of Next Generation Sequencing (NGS) technologies and the concomitant reduction in sequencing costs allows unprecedented high throughput profiling of biological systems in a cost-efficient manner. Modern biological experiments are increasingly becoming both data and computationally intensive and the wealth of publicly available biological data is introducing bioinformatics into the “Big Data” era. For these reasons, the effective application of High Performance Computing (HPC) architectures is becoming progressively more recognized also by bioinformaticians.

Here we describe HPC resources provisioning pilot programs dedicated to bioinformaticians, run by the Italian Node of ELIXIR (ELIXIR-IT) in collaboration with CINECA, the main Italian supercomputing center.

**Results:**

Starting from April 2016, CINECA and ELIXIR-IT launched the pilot Call “ELIXIR-IT HPC@CINECA”, offering streamlined access to HPC resources for bioinformatics. Resources are made available either through web front-ends to dedicated workflows developed at CINECA or by providing direct access to the High Performance Computing systems through a standard command-line interface tailored for bioinformatics data analysis. This allows to offer to the biomedical research community a production scale environment, continuously updated with the latest available versions of publicly available reference datasets and bioinformatic tools. Currently, 63 research projects have gained access to the HPC@CINECA program, for a total handout of ~ 8 Millions of CPU/hours and, for data storage, ~ 100 TB of permanent and ~ 300 TB of temporary space.

**Conclusions:**

Three years after the beginning of the ELIXIR-IT HPC@CINECA program, we can appreciate its impact over the Italian bioinformatics community and draw some considerations. Several Italian researchers who applied to the program have gained access to one of the top-ranking public scientific supercomputing facilities in Europe. Those investigators had the opportunity to sensibly reduce computational turnaround times in their research projects and to process massive amounts of data, pursuing research approaches that would have been otherwise difficult or impossible to undertake. Moreover, by taking advantage of the wealth of documentation and training material provided by CINECA, participants had the opportunity to improve their skills in the usage of HPC systems and be better positioned to apply to similar EU programs of greater scale, such as PRACE. To illustrate the effective usage and impact of the resources awarded by the program - in different research applications - we report five successful use cases, which have already published their findings in peer-reviewed journals.

## Background

The amount of data generated by various scientific experimental platforms is growing exponentially with modern high-throughput technologies. In the case of life science, with the advent of new Next-Generation Sequencing (NGS) technologies [1] and their many applications (e.g, [2–5]), we witnessed to what is defined as a biological data deluge [6]. This large data flux introduces de-facto bioinformatics into the “big data” era [7], however the analysis and interpretation of these massive amounts of data entails relevant computational challenges.

The number of research groups working with NGS data has grown continuously in the last few years and, accordingly, their needs in terms of computational and storage facilities are constantly increasing. It’s becoming more and more common that the hardware requirements needed to perform a bioinformatic analysis in a convenient frame of time far exceed those available in desktop computers, servers, and even local IT facilities. As a result, biologists and bioinformaticians are increasingly making use of HPC architectures made available by supercomputing centers [8].

High performance computing availability is thus becoming a key factor in making effective use of high throughput sequencing (HTS) biological data, that is extracting meaningful biological information in a^1^ reasonable amount of time. The typical scenario involves biological datasets that can easily include billions or even trillions of NGS reads and hundreds or thousands of samples, involving a storage occupancy size up to hundreds of TB per experiment or research project (e.g. MEGA, the European Alliance for Personalized Medicine). Furthermore, most use cases require complex analytical workflows implying the execution of multiple steps and the usage of several- open source or proprietary- bioinformatics tools. The number of samples included in an analysis can be further increased by the re-analysis of similar experiments previously conducted by other research groups and accessible through web archives storing HTS data (e.g. The Sequence Read Archive SRA^1^, the Cancer Genome Atlas TCGA,^2^ the Genotype-Tissue Expression GTex^3^ among others) in order to compare and validate the results obtained.

However, it must be also noted that the majority of currently available bioinformatics tools are not yet developed nor optimized with HPC in mind, therefore their availability on generic HPC facilities is still limited. As a consequence, most life science researchers and bioinformatics software users and developers are not used to interact with public HPC facilities and make a limited use of the ones available to them. Offering an HPC service tailored for bioinformatics analyses can therefore provide relevant advantages that go beyond the possibility of analysing massive amounts of biological data in a timely manner. In fact, it introduces life science researchers to the HPC work environment, raising their awareness of the opportunities offered by HPC and thus making more likely the future development of tools, algorithms, applications and implementations optimized for HPC environments possibly stemming a positive feedback loop.

## Implementation

### The ELIXIR-IT HPC@CINECA call: HPC resources for the bioinformatics community

ELIXIR-IT constitutes the Italian Node of ELIXIR, an intergovernmental organisation that brings together life science computational infrastructures and resources from across Europe with the objective of creating a homogenous and seamless infrastructure for biological data. ELIXIR-IT is structured as a Joint Research Unit (JRU) named ELIXIR-IIB (Infrastruttura Italiana di Bioinformatica - Italian Infrastructure for Bioinformatics), and currently involves 23 partners among Universities and Research Institutions/Facilities of national relevance and led by the National Research Council (CNR).

On april 2016, ELIXIR-IT and CINECA, one of the founding members of the JRU, launched a pilot project named ELIXIR-IT HPC@CINECA, aimed at providing an entry-level -but still substantial- package of HPC resources (50 k core hours, 1 TB of permanent storage extensible depending on the project needs) to research projects presented by Italian and European life science researchers. Three years after its inception, we can evaluate the impact of this initiative that, with over 60 project proposals submitted, an acceptance rate of about 90% and several publications made possible by the HPC resources assigned, can now be considered as a successful experimental service.

Project proposals are submitted to HPC@CINECA through a simple dedicated online form^4^, where the applicants are required to describe relevant aspects of their project including the motivations that make access to an HPC facility convenient. Subsequently, a technical and scientific evaluation committee (see below), evaluates the project to assess its technical feasibility and scientific soundness. Upon approval, the successful applicant gain access to the CINECA infrastructure either i) through a command-line interface (for those who need flexibility and customizable components) or ii) through a set of predefined web pipelines (for those who are not confident with command line and identify in such packaged solutions valid tools for their analyses). If needed, users can also request the installation of specific software or reference dataset not yet available on the CINECA HPC bioinformatic environment. All the new software is installed on the system by an expert support team and made available to all the users of the HPC platform. This mechanism helps in keeping the CINECA HPC work environment continuously up-to-date with the most widely used software tools and reference datasets (for further details refer to the file software.xlsx in the supplementary materials).

**Fig. 1:**
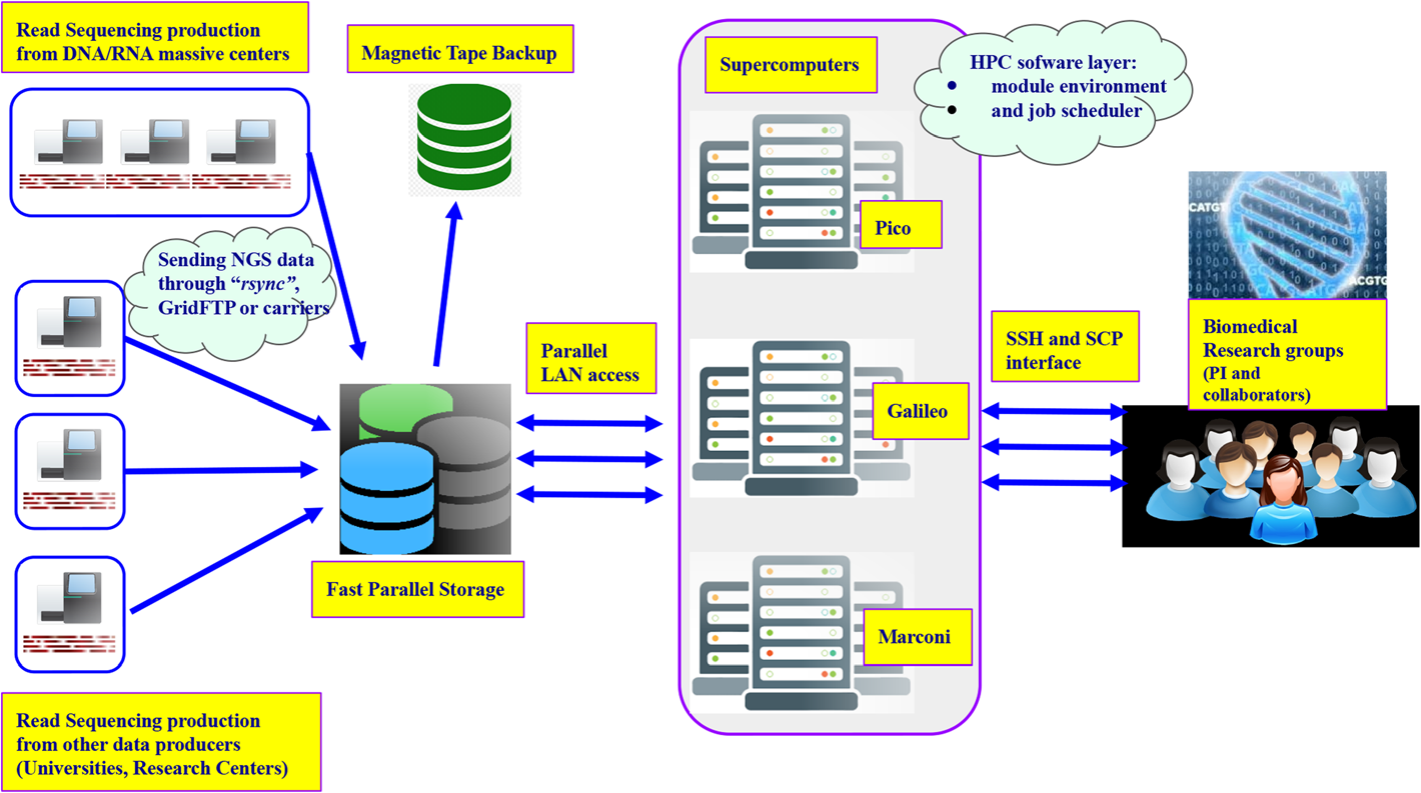
The typical flow for novel NGS data transferred to CINECA and analysed by ELIXIR-IT HPC@CINECA users. The CINECA storage facility provides iRODS [9] technology to archive and facilitate moving data between different supercomputers if needed

The transfer of local or remote data to the CINECA storage facility can be done either using: (i) Rsync or GridFTP exploiting the CINECA Intel QDR (40Gb/s) Infiniband high-performance network, for those sources with sufficient bandwidth, or (ii) sending by carrier portable storage devices to the CINECA user support team for those users with insufficient bandwidth. A schematic of this process is represented in Fig. 1.

### CINECA supercomputers facility

CINECA continuously invests in state-of-the-art resources for computing and storage, usually opting for general purpose hardware compatible with the widest possible range of scientific domains. For this reason, the hardware setup (Table 1) of the computational environment available to ELIXIR-IT HPC@CINECA users has slightly changed over the years, mostly in order to provide the best possible available solutions. At the time of writing, HPC@CINECA users have access to two supercomputers, Marconi (Tier 0) and Galileo (Tier 1), available for production runs. Which one is assigned to each user depends on the resources required by the project. A third legacy cluster called Pico (Tier 1), which was the system initially reserved for the HPC@CINECA program back in 2016, is now reserved only to projects with particular high demands in terms of random access memory.

**Table 1:** Cineca high-performance computing clusters available for bioinformatic projects during the ELIXIR-IT HPC@CINECA call period, some providing higher-memory nodes. Projects are assigned to one cluster or the other upon technical evaluation from Cineca’s staff and depending on the nature and needs of the project itself. Detailed instructions on how to get access to and use the clusters are provided to the PIs after projects approval and are also available on the Cineca website. Marconi A2 is going to be replaced by the Marconi 100 cluster in a few months

HPC cluster	Nodes	Total core	RAM/Node (GB)	Architecture
Pico (2015–2017)	70	1400	128	*Intel Sandy-Bridge*
Galileo	516	16,512	128	*Intel Haswell + GPU*
Marconi A2	3176	215,968	96 (cache mode)	*Intel Knights Landing*

A more thorough description of these computational platforms is available from the CINECA portal.^4^The CINECA data storage facility consists of arrays of high throughput devices (based on the GSS technology) for a total amount of about 4 PB of storage, connected to a large capacity tape library for a total actual amount of 12 PByte (expandable to 16 PByte).

**Fig. 2:**
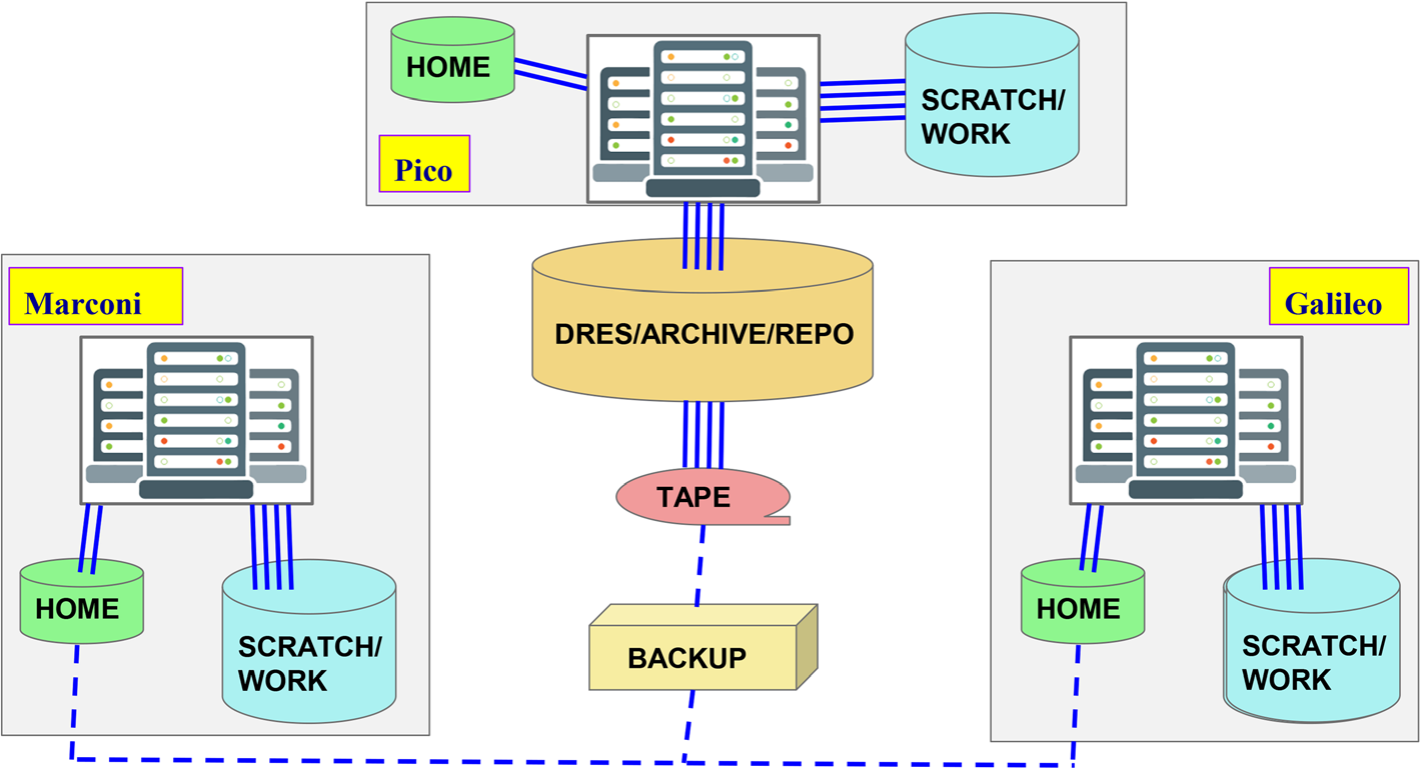
Schematic draw of CINECA system infrastructure . (i) HOME area: intended for source codes, executables, small data files; (ii) SCRATCH area: intended for the output of batch jobs; (iii) WORK area: output of batch jobs as well as for secure sharing within the project team; (iv) DRES: intended as a medium/long term repository and as a shared area within the project team and across HPC platforms; (v) tape area: i personal long term archive area - via Linear Tape File System (LTFS)

All CINECA supercomputers use a common file system layout: shared areas complement the usual local HOME, SCRATCH and WORK private storage areas intended respectively for executables, big outputs and final outputs shared with the rest of the project team. A schematic and more detailed description of the file system of each cluster is reported in Fig. 2.

### Command line software environment

A typical bioinformatics analysis pipeline requires the execution of complex workflows that can be logically represented as modules organized in a graph. These workflows often integrate several custom scripts as well as open source bioinformatics tools. Since software packages installation and maintenance can require considerable efforts by the user, the HPC@CINECA support team provides bioinformatics software within the CL environment through the “Environment Modules” UNIX package [10]. This solution allows users to easily load and unload tools, reference genomes and annotations required for their analyses within their own working environment. Table S1 reports the complete portfolio of currently available bioinformatics software for HPC@CINECA users. Users can always require the installation of additional software system-wide or add, on their own, software and custom scripts to their account.

Furthermore, each cluster currently incorporates reference datasets consisting of reference genome assemblies and annotation files for model organisms such as human (hg18, hg19, hg38); mouse (mm9, mm10); cow (bostau7, bostau8); horse (EquCab3); pig (*Sus scrofa* 11.1); rat (rn6) an so on, as well as a collection of meta-data for major commercial exome kits (Illumina, Agilent and Nimblegen).

At the time being the software environment is not GDPR compliant and therefore project applications involving the analysis of sensitive data have to be turned down.

### Scientific workflows

ELIXIR-IT HPC@CINECA provides access also to fully automated, expert designed and highly optimized bioinformatics pipelines which allow to perform routine NGS data analysis through user-friendly web interfaces. Three different analysis workflows are currently available:

1. CoVaCS [11], a fully automated system for genotyping and variant annotation of resequencing data produced by second generation NGS technologies. CoVaCS offers state of the art tools for variant calling and annotation along with an expert made pipeline for the analysis of whole genome shotgun (WGS), whole exome sequencing (WES) and targeted resequencing data (TGS), performing all steps from quality trimming of the sequencing data to variant annotation and visualization. The final set of variants is obtained by forming a consensus call-set (2 out of 3 rule) from three different algorithms based on complementary approaches: Varscan, GATK and Freebayes. The system is currently available at this URL.^5^

2) RAP [12] a web interface based package that allows the execution of several operations on RNA-Seq data, including: quality control, alignment, abundance estimation and differential expression analysis at gene and transcript levels, differential alternative splicing and polyAdenilation, detection of fusion transcripts. The web interface of RAP is available at this URL.^6^

3) Expedit [13] is a web-service application dedicated to the exploration of RNA editing from human RNA-Seq data at preset or user-supplied specific genomic coordinates. Input data can be provided both in the form of raw reads (FASTQ or SRA format files) files, or in the form of aligned reads (in SAM/BAM format). The comparative analysis is carried on against a large collection of known editing sites collected in the DARNED database [14] as well as other user-provided potentially edited positions. Final results are displayed as custom tracks at the University of California, Santa Cruz (UCSC) genome browser, for a quick examination of the genomic context. ExpEdit is freely available at the following link.^7^

### Evaluation of the project proposals and user support

Project proposals submitted to the ELIXIR-IT HPC@CINECA program are initially evaluated by a scientific and technical board, appointed by ELIXIR-IT, to ensure the scientific soundness and technical feasibility of the application.

Projects are evaluated with a “first come, first served” policy until the total annual resource budget of the program, consisting of over 1 M core hours, has been assigned. Applications are evaluated within seven working days from submission, while approved applications obtain access to the computational resources within further seven working days from acceptance.

Users associated to a project are classified either as principal investigator (PI), typically who submitted the project proposal, or as part of a PI-managed set of authorized users. The standard package allocated to each approved project consists of 50 k core hours and 1 TB of permanent storage, for a duration of 6 months, extensions of the projects deadline and requests of additional resources are considered on a case-by-case basis taking into account their scientific merit.

The jobs submission procedure makes use of the free and open-source job scheduler SLURM, a computer application for controlling unattended background program execution of jobs. Jobs requiring more than 10 min of core/hours execution need to be executed exclusively using SLURM directives. The job scheduler of the CINECA’s HPC multi-user environment has been configured so that each user is allowed no more than 20 jobs at the same time. The core hours employed by users associated to a project are, in turn, subtracted from the core hours budget of the project itself.

To help users dealing with the complexity of the command line interface and of the job scheduler, CINECA makes available a user support team always readily available to help researchers in:

- Setting up and enriching the software environment, with the quick deployment of any additional module required by a project;
- Automating the analysis of large amounts of data, with fine-tuned, cluster-specific, configuration of parameters for the different platforms;
- Helping in optimizing the analysis of big-data experiments;
- Collaborating in the development of highly parallelized code (through MPI, python, R, etc.) suitable for HPC infrastructures;
- Automating the bioinformatics pipelines via SLURM directives.

## Results

**Fig. 3:**
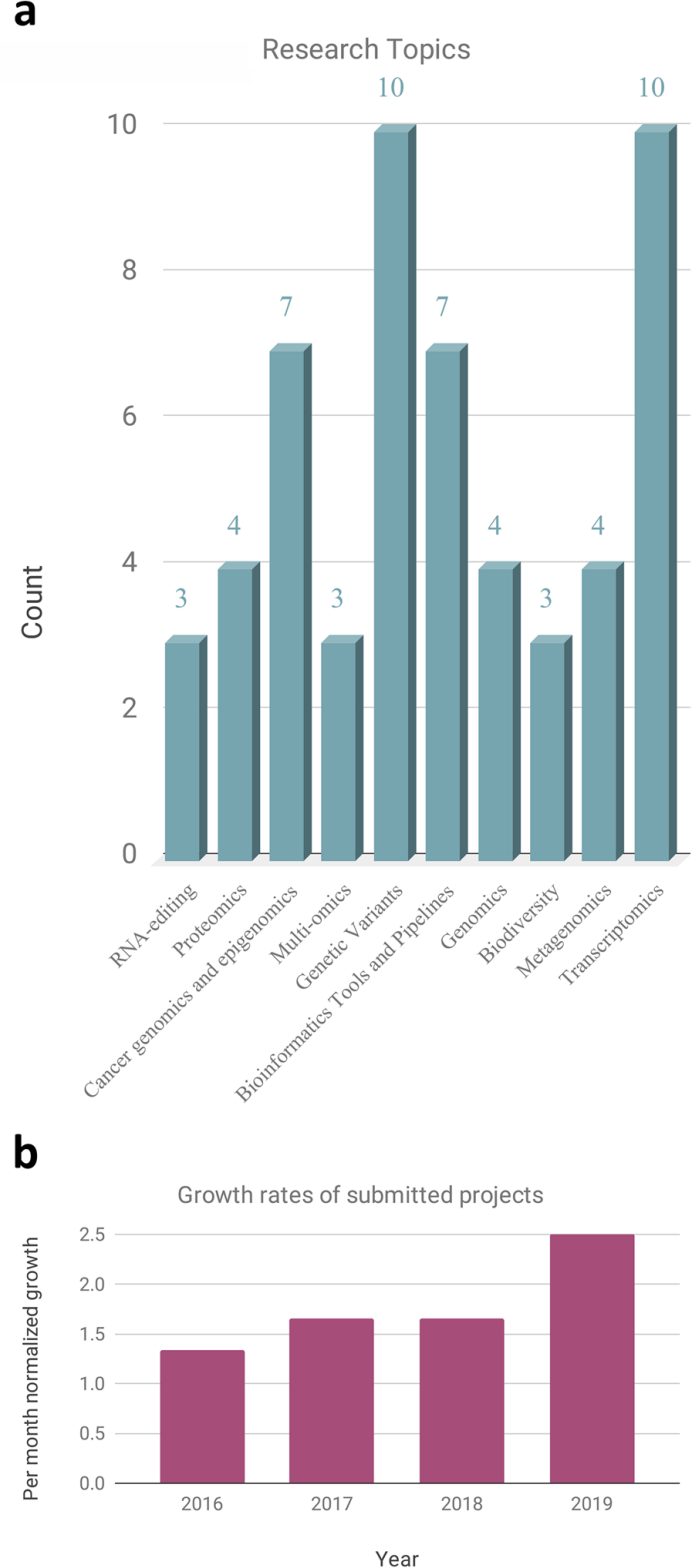
Panel A) show the distribution of research projects between broadly-defined research areas Panel B) shows the growth rates of submitted projects normalised per month, only 4 months were considered for 2016 (starting year) and 2019 (current year).

Since its launch on april 2016, ELIXIR-IT HPC@CINECA has provided access to HPC resources to 63 research projects, allocating a total of approximately 3,250,000 CPU core hours. The 63 projects are distributed among 28 research centres (Universities and Research Centers). Figure 3a reports the distribution of proposed projects over several biological macro-areas, showing as the initiative engaged researchers from different backgrounds and different needs in terms of computational requirements and software. As shown in Fig. 3b, the number of projects submitted is growing constantly since the first opening of the call. The growth can be ascribed both to dissemination activities performed in several national conferences [15, 16] as well as to the good feedback obtained from the first participants, as demonstrated by the publication of the results of HPC@CINECA research projects in peer reviewed scientific journals.

### Use cases

Here we provide a brief summary of some research projects that were successfully completed thanks to the HPC resources provided by the ELIXIR-IT HPC@CINECA call.

#### Genome-wide mapping of 8-oxo-7,8-dihydro-2′-deoxyguanosine across human genome

8-Oxo-7,8-dihydro-2′-deoxyguanosine (8-oxodG) is one of the major DNA modifications that occurs when the DNA is exposed to pro-oxidant species (ROS) generated by endogenous metabolism. 8-oxodG is a potent premutagenic lesion for its ability to pair with both cytosine and adenine residues, thus causing G:C to T:A transversions during DNA replication [17, 18].

Several thousand residues of 8-oxodG are constitutively produced in the genome of mammalian cells and a new method has been developed to identify their genomic distribution.

Recently, by using OxiDIP-Seq, Amente et al. [amenteetaloxidip19] reported the genome-wide distribution of 8-oxodG in proliferating DDR-proficient mammary cells (MCF10A and MEFs). Analysis of OxiDIP-Seq revealed that endogenous 8-oxodG is regioselective distributed across the mammalian genome. Moreover, an integrated data analysis starting from OxiDIP-Seq, ChIP-Seq anti-gH2AX, ChIP-Seq anti-POLII, GRO-Seq and RNA-Seq led to the identification of an accumulation of endogenous DNA damage within the gene body of long genes with poor-to-moderate transcription levels. In terms of computational resources we used 2 TB of permanent storage and 200 k core/hours to perform all the analysis of about 500 GB of starting input data. They were analyzed using HPC@CINECA computer resources,) through both the command line environment and the bioinformatics automated pipelines [refs. To RAP and CAST] developed and provided by the CINECA-ELIXIR IT team. This computational effort led to further insights about the molecular mechanisms underlying the heterogeneity of the local mutation rate and the understanding of why certain regions seem to be more, while others less, prone to oxidation. A full description of this work can be found in [19].

#### New HPC-optimized algorithm for prediction of RNA-editing events from RNA-Seq data

RNA editing is a relevant epitranscriptome modification occurring in a wide range of organisms. In humans, it affects nuclear and cytoplasmic transcripts mainly by the deamination of adenosine (A) to inosine (I) through ADAR enzymes acting on double RNA strands [LiChurch13]. RNA editing has a plethora of biological effects and its deregulation has been linked to a variety of human diseases including psychiatric, neurological and neurodegenerative disorders, and cancer [20]. Several bioinformatics tools to investigate RNA editing events in NGS data have been released [21]. However, its computational identification is a highly time-consuming process, requiring the traversing of very large alignments files in BAM format, position-by-position. Employing ELIXIR-IT HPC@CINECA resources the original REDItools package [22], one of the most accurate tools to call RNA editing events in RNA-Seq experiments [21], the A-to-I calling process has been speeded up, optimizing its implementation for HPC infrastructures:

- a first optimization in the new version of the code, REDItools2.0, consisted in loading the sequences from disk by reading each sequence only once, keeping it in memory until no longer than needed. This implementation was on average 8–10 times faster than the original version running on a single core;
- another improvement of the algorithm consisted in optimizing the splitting of the genome into genomic intervals. The initial release of REDItools treated equally different chromosomal regions, by dividing the whole genome in chunks of equal size and assigning each chunk to a thread. Since usually expression data do not exhibit a constant coverage, the number of reads per genomic unit (density of mapped reads) is quite variable and the original version of REDItools spent a lot of computational time in high-density genomic regions. We therefore implemented an optimal interval division in order to guarantee an approximately uniform per-thread workload;
- a parallel version of REDItools2.0 has also been implemented by writing an ad-hoc MPI Python script based on the use of the mpi4py library [23]. This library provides binding of the Message Passing Interface (MPI) standard for the Python programming language. In this way it is possible to exploit multiple computing nodes by means of collective communication MPI primitives. A simple master/slave template has finally been implemented for coordinating the overall computation.

**Fig. 4:**
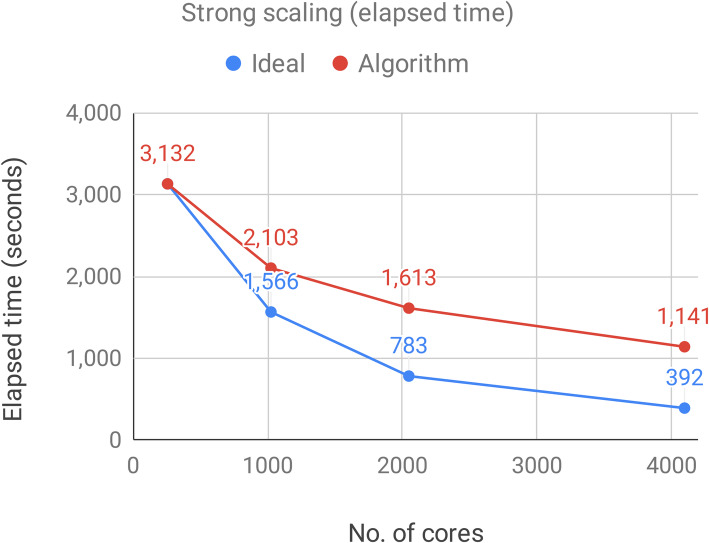
Evaluation of the scalability of the optimized REDItools version on the Marconi-A2 infrastructure using 540, 1080, 2160 and 4320 cores. The plot shows the elapsed time (in seconds) needed to analyze a single sample when using an increasing number of cores

Executions of the optimized algorithm on real RNA-Seq experiments have shown that the novel REDItools2.0, is on average ten times faster than the previous implementation and the speed up scales adequately with the number of cores involved in the analysis (Fig. 4) thus representing the first HPC resource specifically devoted to RNA-editing detection.

Thanks to the algorithmic optimization described above, the novel REDItools2.0 package has been then used to investigate RNA editing in very large cohorts of RNA-seq experiments like those produced in GTEx or TCGA projects after the award of additional resources through a competitive PRACE (Partnership for Advanced Computing in Europe) project (ProjectID: 2016163924 GREaT - Genome wide identification of RNA editing sites in very large cohorts of human whole transcriptome data). Full description of this work is available in *PRACE White Paper.*^8^

#### Creation of a comprehensive database for genomics data in peach (*P. persica* L. Batsch)

Peach is an economically important fruit tree species of temperate region. Integrating novel genomics tools is a fundamental goal for increasing the efficiency of breeding activities and the leveraging of basic knowledge in this species. After the release of the first peach genome draft, the remarkable advances in high-throughput molecular tools has led to the generation of a multitude of genomics data from several whole-genome re-sequencing projects.

In this project, Whole-genome sequencing data of 125 peach (*P. persica* L. Batsch) accessions and 21 wild relatives of the Amygdalus subgenus have been downloaded from the NCBI SRA [24] for a whole of 146 accessions publicly available (input data size about 10 TB). Variant discovery was achieved by applying an imputation-free joint variant-calling procedure on the 146 accessions, improving variant discovery by leveraging population-wide information from a cohort of multiple samples [25]. 200 k core/hours have been used to analyse all the samples on the Pico cluster to create the compendium dataset of peach variants. The identified peach variants, both SNP and InDels, are available at the PeachVar-DB portal^9^ that provides an easy access to the information mined from peach Whole Genome Re-Sequencing (WGRS) data. Full description of this work can be found in [26].

#### High-quality genome assembly for the European barn swallow (*Hirundo rustica rustica*)

The barn swallow is a passerine bird with at least eight recognized subspecies in Europe, Asia, and North America. Due to its synanthropic habits and its cultural value, the barn swallow is also a flagship species in conservation biology [27]. The availability of high-quality genomic resources, including a reference genome, is thus pivotal to further boost the study and conservation of this species. To facilitate further population genetics and genomic studies, as a part of the Genome10K effort on generating high-quality vertebrate genomes (Vertebrate Genomes Project) [28].

Formenti et al. [29] have assembled a highly contiguous genome assembly using single molecule real-time (SMRT) DNA sequencing and Bionano optical map technologies for the European subspecies (*Hirundo rustica rustica*). The assembly of the genome, which was performed entirely on the Marconi CINECA HPC supercomputer occupied 3840 central processing unit (CPU) hours and a total amount of 2.2 Tb of random access memory (RAM) for reads correction, 768 CPU hours and 1.1 Tb of RAM for the trimming steps, and 3280 CPU hours and 2.2 Tb of RAM for the assembly phase. The entire process was completed in less than 5 days on the CINECA HPC platform, while re-analysis of the same data on a local server required more than 80 days (Matteo Chiara, personal communication) at full computational capacity.

After removal of haplotigs, the final assembly resulted in approximately 1.21 Gbp in size, with a scaffold N50 value of more than 25.95 Mbp, representing a considerable improvement over the previously reported assembly [30]. Systematic comparisons of this high quality draft genome assembly of *H. rustica* with a collection of closely and distantly related bird genomes provide phylogenomics profiles of structural rearrangements and gene losses/gene duplications. The approach used for the assembly of the barn swallow genome, while attesting to the effectiveness of SMRT sequencing combined with DLS optical mapping for the assembly of vertebrate genomes, provides an invaluable asset for population genetics and genomics in the barn swallow and for comparative genomics in birds. Full description of this work can be read in [29].

#### Massive NGS data analysis reveals hundreds of potential novel gene fusions in human cell lines

One of the genetic alterations that are linked to cancer development in addition to single nucleotide mutations are gene fusions deriving from chromosome rearrangements. The availability of sequence data from NGS techniques has made possible the discovery of a huge amount of such alterations. However, current algorithms for fusion detection either have high false positive result rates or miss some real events. Hence, it is very important to be able to run and compare the results of several algorithms, with different discovery properties.

Gioiosa et al. [31] have extensively carried out the analysis of 935 paired-end RNA-sequencing experiments downloaded from the Cancer Cell Line Encyclopedia repository (CCLE),^10^ for a total of 32 TB of input raw data. The aim was addressing novel putative cell-line specific gene fusion events in human malignancies. Four gene fusion detection algorithms were launched on the CCLE samples to detect gene fusion events, for a total of 500 k core/hours. Furthermore, a prioritization analysis was performed by running a Bayesian classifier that adds an in silico validation on detected events. The collection of fusion events supported by all of the predictive algorithms provides a robust dataset of ∼1700 in silico novel candidates among gene fusion events. These data have been stored, collected and integrated with other external resources within the LiGeA portal (cancer cell LInes Gene fusion portAl),^11^ where they are browsable and freely downloadable. Full description of this work can be found in [31].

## Discussion

### HPC perspectives for the bioinformatics community

CINECA is strongly focused on understanding and collecting all the needs of researchers in the bioinformatics field. Besides the HPC systems involved in the ELIXIR-IT HPC@CINECA pilot call, multiple prototype systems are provided to be tested with new test cases. For example, systems with hierarchical memory equipped with fast high bandwidth memory (HBM), standard DDR4 RAM and newly available non-volatile memory (NVM). The latter is especially interesting for bioinformatics test cases since it can provide cost effective solutions with TBs of RAM memory [MC1] with performance comparable with standard DDR4. Challenging bioinformatics problems that can benefit from the use of High-Performance computational resources requiring tremendous RAM availability regards:

- metagenomics research in which scientists need to analyze over a million metagenomes in search of signature genes that could serve important functions in biomanufacturing for health, energy, and industry.
- large genome assembling with PacBio single molecule sequencing reads.
- analysis of large cohorts of individuals (> 10,000 samples) with Genome Wide Association Study (GWAS) techniques.

**Fig. 5:**
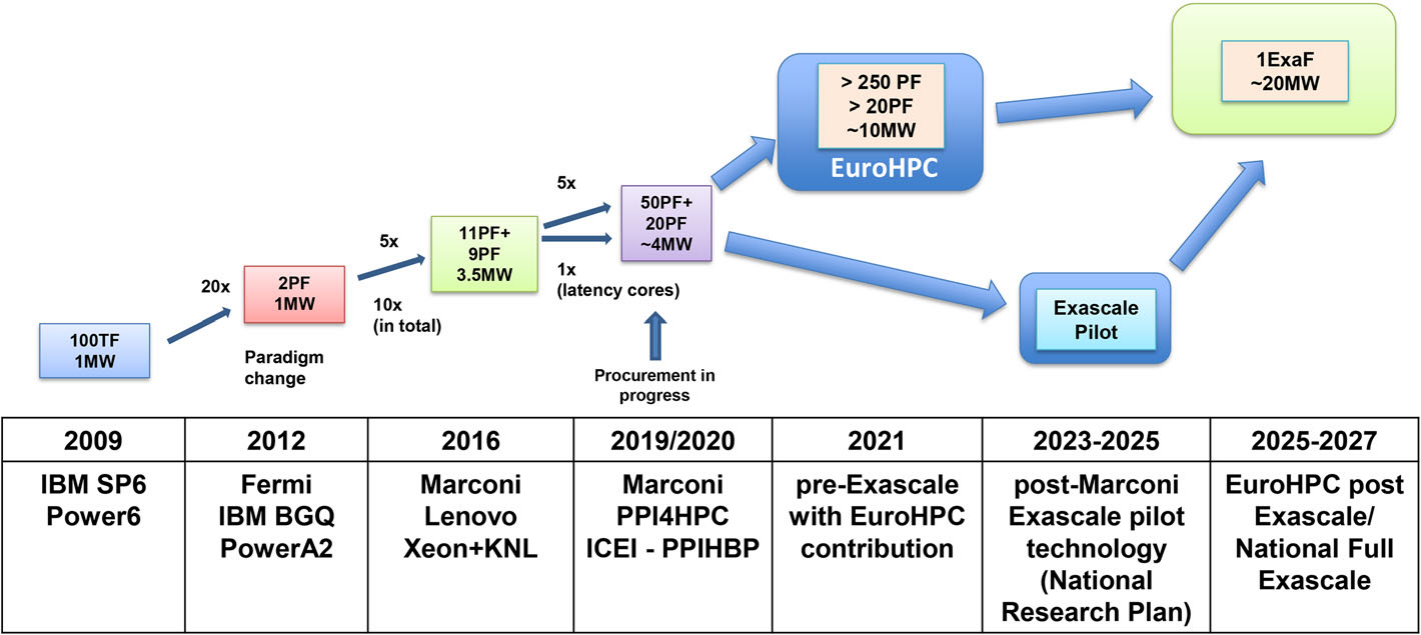
CINECA roadmap towards systems of exaflop capabilities

CINECA will also devote considerable efforts in keeping the HPC infrastructure up to date with the most advanced technological solutions that will become available in the forthcoming years. In this regard, Fig. 5 shows the roadmap of future development of CINECA HPC systems in the timeframe 2020–2027: different routes may be taken but the clear goal is to provide researchers with a world class exascale HPC systems by 2025–2027. In particular, pre-exascale systems may be available as early as 2020 through the EuroHPC European initiative [32] or alternatively, in the following years, through Italian national funding. A typical computationally demanding application, which could potentially benefit from exascale architecture, could be represented by personalized medicine, since in this field the celerity of data analysis can be crucial for saving people’s lives.

Indeed, computational challenges for personalized medicine usually pursue the objectives of:

- providing systems for handling and analysing heterogeneous patients’ data and clinical questions, for example through the implementation of new parallel deep learning algorithms;
- developing optimized, secure and law-compliant personalized medicine computational pipelines;
- modelling and predicting drug response, understanding the molecular basis of key cellular interactors and speeding up the extraction of information from millions of disease patient records to determine optimal disease treatment strategies;
- producing and testing all the components necessary to exploit Exascale calculation to generate patient models.

Moreover, a convergence between traditional HPC and artificial intelligence (AI) is planned in the first Exascale platform to be installed in the USA at the Argonne National Laboratory in 2021. Bioinformatic is going to fully take advantage of this convergence, with new applications not only in personalized medicine but in the full range of molecular biology research.

From the user standpoint, the increase in computational power has to match the usability of the systems. To this extent, it is clear the importance of the work of CINECA staff described in this paper. We foresee in the future a more data-centric approach in using the HPC resources, where data stored in the system are subject to multiple analysis, possibly involving a range of technological solutions, such as computing accelerators (NVIDIA, AMD GPUs), specific services hosted in virtual machines (OpenStack, Cloud solutions), fast and big-mem memory nodes (HBM, NVM), and many others.

## Conclusions

In this paper, we describe a pilot program for provisioning HPC resources to bioinformatics researchers with the aim of providing a viable solution for large scale analyses of massive NGS data, a need that is becoming increasingly common in the biomedical research community.

At the beginning of massive sequencing advent, the analysis software used only a single core and no parallel programming techniques, such as threading or MPI. This has changed over the years and many current applications take advantage of the multi-threading modality in order to accelerate the calculations.

In summary, having a good mix of general purpose computational power, the possibility of booking an arbitrary number of cores (multi-threading) or nodes for the jobs that require more CPUs, or very large memory nodes for jobs with large memory requirement, has been shown to be a very efficient and affordable infrastructure for bioinformatic data analysis. As a confirmation of this trend, four out of the five success stories reported in the “Result” paragraph (*a,c,d,e* test cases) were optimized on cluster machines by using a multi-threading approach for most of the programs used by the applied pipelines, and MPI optimization was used in the remaining test case (*b*).

Currently the ELIXIR-IT HPC@CINECA program constitutes, at the best of our knowledge, a unique initiative in the bioinformatics landscape since it is open to small-to-medium sized projects from all European life science researchers based only on the technical and scientific soundness of their applications. Usually, the access model to HPC resources for bioinformatics is bound to serve researchers from specific institutions or collaborating to specific projects, or require researchers to win competitive calls, like PRACE, usually reserved to projects of much greater scale (millions of core hours per project). Actually, the utility of the program has been not only to allow the execution of complex bioinformatic analyses, but also to provide its maintainers with insights on how to organise and manage one of the few public High Performance Computing platforms dedicated specifically to bioinformatics in Europe, and the only one of this type available in Italy. Since the needs of the bioinformatics community in terms of computational and storage resources in the coming few years will rise steadily, this initiative can represent a valid model for the provisioning of HPC services also by other ELIXIR Nodes or similar infrastructural entities in the near future. As one of the key technological partners of ELIXIR-IT, CINECA continuously explores possible actions to improve the technological infrastructure and provide valuable computational services to European life scientists and bioinformaticians. On their part, ELIXIR-IT and CINECA will continue their commitment to this initiative, possibly expanding it with new features and integrating the HPC platform with other services.

### Availability and requirements

Project name: ELIXIR-IT HPC@CINECA.

Project home page: http://www.beaconlab.it/HPC-cineca

Operating system(s): Platform independent.

Programming language: Not applicable.

Other requirements: SSH client.

License: Not applicable.

Use of the platform is subject to approval (see http://www.beaconlab.it/HPC-cineca**).**

## Supplementary information

**Additional file 1 Table S1**: list of the bioinformatics software available for HPC@CINECA users on the three computational platforms (PICO, MARCONI and GALILEO) updated to May 2019. The software portfolio reflects the needs of the users since 2016 and is gradually enriched to meet the requirements of newly approved projects.

## Data Availability

Not applicable.

## Abbreviations

NGS: Next Generation Sequencing
HPC: High Performance Computing
ELIXIR-IT: The Italian Node of ELIXIR
HTS: High throughput sequencing
JRU: Joint Research Unit
WGS: Whole genome shotgun
WES: Whole exome sequencing
TGS: Targeted genome re-sequencing
PI: Principal investigator
8-oxodG: 8-Oxo-7,8-dihydro-2′-deoxyguanosine

## Footnotes

1 https://www.ncbi.nlm.nih.gov/sra Accessed 18 Mar 2020

2 https://tcga-data.nci.nih.gov Accessed 18 Mar 2020

3 https://commonfund.nih.gov/GTEx Accessed 18 Mar 2020

4 https://wiki.u-gov.it/confluence/display/SCAIUS/HPC+User+Guide Accessed 18 Mar 2020

5 https://bioinformatics.cineca.it/covacs Accessed 18 Mar 2020

6 https://bioinformatics.cineca.it/rap/ Accessed 18 Mar 2020

7 https://bioinformatics.cineca.it/expedit/ Accessed 18 Mar 2020

8 https://prace-ri.eu/wp-content/uploads/WP258.pdf Accessed 18 Mar 2020

9 http://hpc-bioinformatics.cineca.it/peach/main Accessed 18 Mar 2020

10 https://portals.broadinstitute.org/ccle Accessed 18 Mar 2020

11 http://hpc-bioinformatics.cineca.it/fusion/ Accessed 18 Mar 2020
